# Capacitance–Voltage Studies on Electrostatically Actuated MEMS Micromirror Arrays †

**DOI:** 10.3390/mi16020157

**Published:** 2025-01-29

**Authors:** Jiahao Chen, Xiaohui Yang, Mustaqim Siddi Que Iskhandar, Md. Kamrul Hasan, Shilby Baby, Muhammad Hasnain Qasim, Dennis Löber, Shujie Liu, Roland Donatiello, Steffen Liebermann, Guilin Xu, Hartmut Hillmer

**Affiliations:** 1 Institute of Nanostructure Technologies and Analytics (INA), Technological Electronics Department and Center for Interdisciplinary Nanostructure Science and Technology (CINSaT), University of Kassel, Heinrich-Plett-Straße 40, 34132 Kassel, Germany; yang@ina.uni-kassel.de (X.Y.); kamrul.hasan@ina.uni-kassel.de (M.K.H.); qasim@ina.uni-kassel.de (M.H.Q.); d.loeber@ina.uni-kassel.de (D.L.); shujie.liu@ina.uni-kassel.de (S.L.); donatiello@ina.uni-kassel.de (R.D.); hillmer@ina.uni-kassel.de (H.H.); 2 Nanoscale Glasstec GmbH, Heinrich-Plett-Straße 40, 34132 Kassel, Germany; mustaqim.iskhandar@nanoscale-glasstec.com (M.S.Q.I.); shilby.baby@nanoscale-glasstec.com (S.B.); s.liebermann@student.uni-kassel.de (S.L.); guilin.xu@nanoscale-glasstec.com (G.X.)

**Keywords:** electrostatic actuation, MEMS micromirror arrays, capacitance–voltage (C-V) measurement, hinge structure, initial tilt angle, pull-in voltage

## Abstract

This article presents the electrostatic actuation performance of micromirror arrays for intelligent active daylight control and energy management in green buildings using a capacitive–voltage (C-V) measurement technique. In order to understand how geometric hinge parameters, initial opening angles, and materials affect the overall efficiency and functionality of the system, micromirror arrays have been analyzed using C-V measurements considering (i) full and broken hinge structures, (ii) 90° and 130° initial tilt angles (Φ), and (iii) different material layer combinations. The measurement results indicate that both an increase in the Young’s modulus of the applied materials and increasing the initial tilt angles increase the threshold voltages during the closing process of the micromirrors.

## 1. Introduction

Over the years, environmental conservation and energy efficiency have consistently been a priority, and advances in science and technology have enhanced our ability to support the environment without altering our lifestyles. These technological strides, visible in various sectors, like renewable resources and energy storage using solar cells, primarily aim to harness natural energy more efficiently, cutting carbon emissions and reducing our reliance on fossil fuels [1]. This paradigm change has become particularly urgent in recent years, intensifying the global pressure to give up fossil energy sources as early as possible. Against this backdrop of heightened environmental awareness and geopolitical pressures, innovative technologies have emerged [2,3,4,5,6]. Our scope lies in the energy transformation of the building sector, of which a substantial portion is energy inefficient [7].

**Figure 1:**
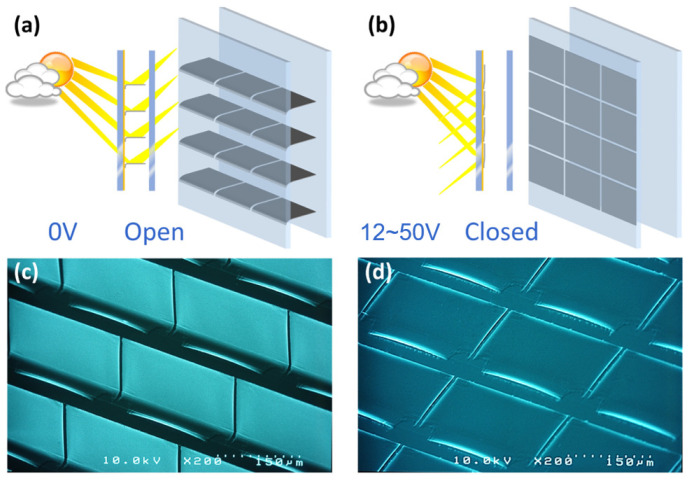
Open state (**a**) and closed state (**b**) of MEMS micromirror arrays, with their respective SEM micrographs in (**c**,**d**). Redrawn from [6].

Due to their dynamic and programmable nature, micromirror arrays in smart windows offer tailored solutions across different scenarios, ranging from residential buildings to high-rise commercial ones [6,8,9]. First, the adjustable capability of micromirror arrays allows for personalized lighting, climatization, and energy management, improving users’ comfort while reducing their reliance on heating, ventilation, air conditioning, and artificial lighting. Second, in high-rise buildings where direct sunlight can cause rooms to excessively heat up, MEMS smart windows can reduce cooling loads and eliminate glare, demonstrating further potential for significant energy savings. Micromirror arrays are mounted in insulation glazing in an inert gas environment, protected from moisture, oxidation, rain, hail and wind. The functionality of MEMS micromirror arrays for smart window applications is shown in Figure 1a, with open micromirrors redirecting daylight into the room when no voltage is applied, and in Figure 1b with fully closed micromirrors when sufficient voltage is applied, thus blocking the daylight from entering the room.

**Figure 2:**
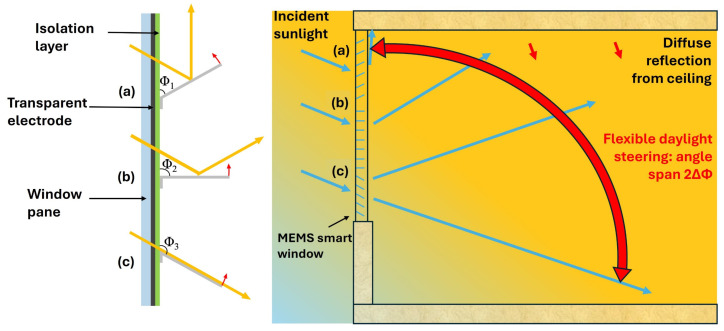
Light steering by different mirror tilt angles (**a**–**c**). For a given daylight incident angle, the light steering span 2ΔΦ is indicated by the red double-arrow and visualized by different blue arrows inside the room.

The state of MEMS micromirror arrays can be adjusted by electrostatic actuation. The different opening states are related to different tilt angles Φ which are controlled by corresponding voltages between the micromirror and a transparent counter-electrode. The adjustment will be made in consideration of the prevailing sunlight conditions to redirect the daylight into the room for the desired personalized light steering. This daylighting functionality not only allows for the adjustment of ambient lighting but also of the room temperature according to the user’s requirements [6,10]. Figure 2a–c shows micromirror arrays with different tilt angles, where daylight is also directed to different parts of the room, as discussed in previous articles [5,6,11,12,13].

In this paper, the initial opening angle of a micromirror is the default angle, where no electrostatic voltage is applied (0 V). The micromirrors in Figure 2a–c are illustrated with tilt angles Φ of ~60°, 90°, and ~130°, respectively, and the same angled light incidence is considered for all three cases. In Figure 2c, involving Φ_3_ in combination with the specified light incidence, no light steering occurs, i.e., the incoming light directly reaches the floor. When the micromirror angle is at Φ_1_, the sunlight is reflected toward the ceiling next to the window. As the angle increases between Φ_1_ and Φ_3_, the reflected light changes over an angle span of 2ΔΦ = 2(Φ_3_ − Φ_1_), indicated by the red double arrow. This further implies the extensive reach of reflected light towards the ceiling area in deeper parts of the room. Such a range and reach demonstrate that the mirror angle span ΔΦ = Φ_3_ − Φ_1_, as illustrated in Figure 2, already covers all the situations required in practice [14,15,16].

**Figure 3:**
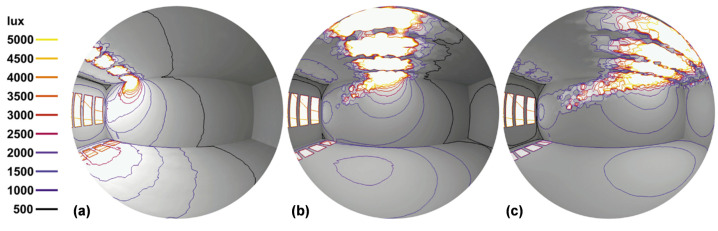
Rendered images in a fisheye lens perspective from inside a model room to display the overall impressions of the illumination distribution. (**a**) Light direction to ceiling parts close to the window. (**b**) Light direction to the middle part of the room ceiling. (**c**) Light direction deep inside of the room. Original from [18], with the permission of Leuze Publishing House.

Rather than the application of this technology in new buildings, the main objective is always to address the issue of energy consumption and energy inefficiency in existing building stock. The incorporation of micromirror array technology in standard insulation glazing production allows for potential energy-efficient retrofitting of existing building stock, particularly high-rise buildings. This is made possible by daylight steering capability and the extended reach to deeper areas of the room. However, an occupant does not use all parts of the room simultaneously. For example, on a hot summer day, the light steering function can provide a personalized light spot at the ceiling above the occupants, enough to provide sufficient ambient lighting, while keeping the rest of the room dimmed and unheated. This can be obtained by subfield addressing using only a small open part of the window, while the rest is kept closed and reflects the heat (see [17] for details). This reduces the required cooling load of the room in summer. By reducing the need for artificial lighting in deeper parts of the room and lowering the demand for air-conditioning (cooling) in summer and heating in winter, significant energy savings can be achieved. Figure 3 shows the distribution of light on the ceiling for different mirror tilt angles [18].

Regarding the application of micromirror smart glass for daylight steering, a large controllable angle span of micromirror arrays is desirable and necessary to achieve a wide range of light steering. In response to different sunlight conditions, the micromirror arrays are supposed to adjust accordingly and subsequently maintain their position at specific tilt angles. As already mentioned, micromirror arrays as a smart window application are fabricated using a subfield addressing technique to provide more flexibility and efficiency for the light steering functionality [17]. The implementation of subfield addressing in the micromirror arrays further stresses the importance of actuation methods and the characterization of their actuation behavior.

Several options exist for MEMS micromirror actuation: electrostatic actuation, electromagnetic actuation, electrothermal actuation, and piezoelectric actuation [19]. First, an electromagnetically actuated micromirror is not considered due to the opacity of permanent magnetic materials, which are not conducive to light transmission [4]. On the other hand, the tilt-angles of piezoelectric actuation are relatively small, and it is preferable to use this technology for high-frequency scanning rather than for quasi-static actuation (fast enough but still always in equilibrium states) [5]. Therefore, electromagnetic and piezoelectric actuation are excluded for our application. The advantages of electrothermal actuation are large forces and deformation outputs, low actuation voltages, and good linearity [20], but the response speeds of electrothermal MEMS are too slow for the smart window [21]. In addition, electrothermal actuation would heat up the window noticeably, adding a definitively unwanted heat source inside the room during the summer months. Considering the advantages of fast actuation speed, low power consumption in large-scale applications, and the inadequacy of other methods to meet our requirement, electrostatic actuation is favored in our application.

Based on capacitance–voltage (C-V) measurements, this paper characterizes 10 × 10 cm^2^ micromirror arrays revealing different designs concerning geometric hinge parameters, initial opening angles, and Ge as a new material. Based on these parameters, the impact on threshold voltages of the closing process and the reopening processes are studied. In Section 2, the fundamental principles of electrostatic actuation are studied along with an illustrative example of a C-V profile. Section 3 details the experimental setups for the C-V measurements, outlining the specific experimental parameters used. In Section 4, C-V profiles for four different designs (A, B, C, and D) are presented and characterized in detail via the C-V technique. These profiles are compared to analyze the impact of the geometric and material parameters. Finally, Section 5 concludes the study with the prospects for future research.

## 2. Principle of Electrostatic Actuation of Micromirror and CV Study

**Figure 4:**
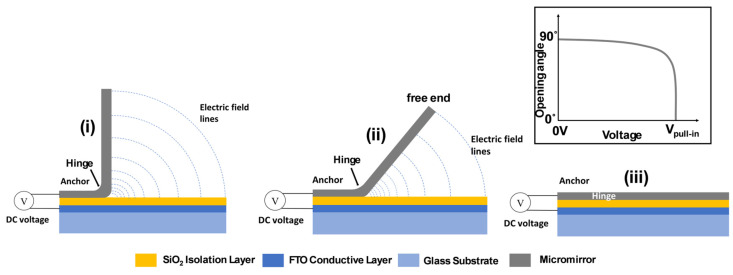
Micromirror at different states: (**i**) the default open state (initial opening angle) when no voltage is applied, (**ii**) the specific tilting position for the chosen applied voltage before the pull-in point, and (**iii**) the closed state. The inset displays the relationship between applied voltage and opening angle considering a special case with an initial opening angle of 90°.

For the actuation of the metallic micromirrors, a DC voltage has been applied between the top and bottom electrodes. The fluorine-doped tin oxide (later FTO) serves as the bottom electrode and the micromirror metal layers act as the top electrode. Both electrodes are separated by a SiO_2_ layer which acts as an isolation layer between these two conductive layers. In Figure 4, the left part of the micromirror is fixed to the substrate and acts as the anchor. The hinge is connecting the planarized mirror area and the anchor. The applied voltage creates an electric field, where the electric field line density is proportional to the local strength of the electrostatic attraction force. They are visualized in Figure 4 as blue dotted lines for three different actuation states of the micromirror.

As shown in Figure 4i, the density of the electric field lines is considerably greater at the hinge area compared to in the mirror areas further away from the hinge. The local electrostatic force is proportional to the density of the electrical field lines; hence the electrostatic attraction force is stronger at the hinge part than at the outer part. These electrostatic forces enable the micromirror to tilt until it completely closes after reaching the pull-in condition. Variations in the electrostatic attraction force can be achieved by changing the applied DC voltage. The moveable mirror plane exhibits various tilt angles depending on the applied voltage. At a low DC voltage, the micromirror is in intermediate state in between (i) and (ii), i.e., slightly tilted, close to the vertical. As the voltage increases, the tilt angle decreases further until the pull-in condition is reached, where the mirror “snaps” onto the SiO_2_ layer and reaches the closed state, as in Figure 4iii [5,6]. The inset of Figure 4 illustrates the relationship between applied voltage (from 0 V until the pull-in voltage) and the tilt angle (from the initial 90° to 0°, describing the closing processes).

Three states are important during the tilt angle variation of the micromirror, including the open state (no voltage applied, here the special case of a vertically standing mirror, Φ = 90°) as shown in Figure 4i. The initial opening angle is defined by residual intrinsic layer stress in the hinge. Figure 4ii depicts an intermediate state, in which the intermediate opening angle can be held by a specific applied voltage between zero and the pull-in voltage. The fully closed state is shown in Figure 4iii, where the applied voltage is equal or larger than the threshold pull-in voltage. At the pull-in voltage, a sudden complete closing of the micromirror occurs (it snaps down towards the isolation layer). The snap-down of micromirrors occurs since a force equilibrium between the electrostatic attraction and the repulsive elastic counter force is no longer possible. The electrostatic attraction force exceeds the restoring elastic force at this pull-in voltage.

**Figure 5:**
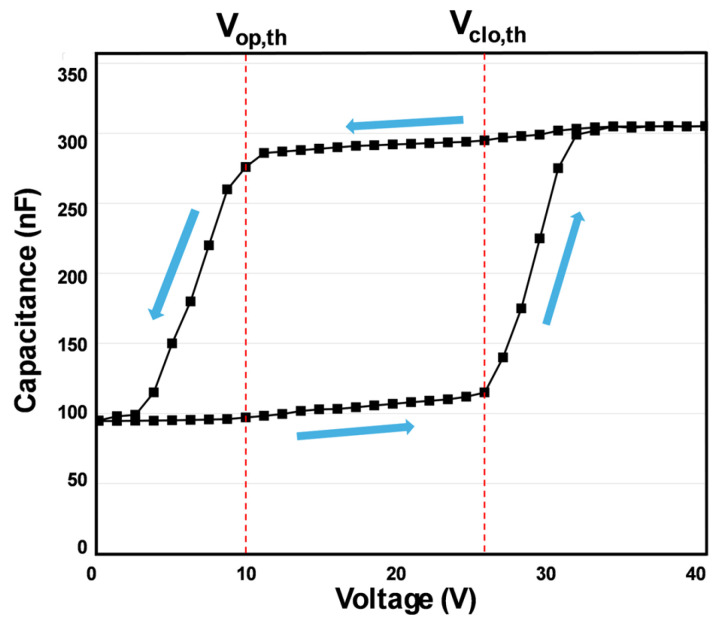
Schematic C-V profile to define the closing and reopening processes and respective threshold voltages. This schematic is obtained by averaging many experimental profiles of different designs. This averaged experimental C-V profile exhibits hysteretic behavior and also reflects the nonlinearities inherent in the actuation mechanism. The threshold voltage of the closing process is V_clo,th_, and the threshold voltage of the reopening process is V_op,th_. The threshold points (threshold voltages) always indicate a transition from a small slope dC/dV to a much larger one. During the closing process, the slopes dC/dV are positive, and during the reopening process, the slopes are negative. The blue arrows show the directions of a full cycle starting from the initial opening angle with the closing process and ending with reopening processes again at the initial opening angle.

In the following, a schematic C-V profile (averaged over many experimental profiles of different designs) is displayed in Figure 5 to define key words, such as the closing and reopening processes and the respective threshold voltages.

The closing process starts at 0 V with the initial opening angle (e.g., 90° or 130°) and ends at the pull-in point (Φ_1_) where the mirrors undergo a sudden snap-in from Φ_1_ to 0°at a certain threshold voltage. The closing process reveals a special threshold point where the small or moderate capacitance increase (slope dC/dV is small and positive) turns into a much larger capacitance increase (slope dC/dV is larger and positive). The threshold voltage of the closing process is abbreviated as V_clos,th_.

The reopening process starts at an angle of 0° and ends at the initial opening angle (e.g., 90° or 130°). The reopening process also reveals a special threshold point where the small or moderate capacitance decrease (slope dC/dV is small and negative) turns into a much larger capacitance decrease (slope dC/dV is larger and negative). The threshold voltage of the reopening process is abbreviated as V_op,th_. The closing and reopening processes reveal a hysteresis (Figure 5).

The relationship between electrode distances and capacitance value in the C-V profile is complex and nonlinear. The function of capacitance versus voltage provides important insights into the device’s performance under various conditions. The combination of these trends altogether manifests the hysteresis of the micromirror arrays actuation mechanism. The interplay between these forces highlights the nonlinear characteristics inherent in MEMS micromirror electrostatic actuation and generates the hysteresis C-V curve, further emphasizing the critical role of these threshold voltages in dictating device actuation (open–closed–open) behavior [22]. The ability to maintain the closed state under reduced voltage conditions not only improves energy efficiency but also contributes to the reliability and long lifetimes of the system in practical applications. Furthermore, the hysteretic nature of this transition highlights the complexities involved in the design and device control, requiring careful consideration of the electromechanical interactions involved in the different applications [23,24,25,26,27,28].

In the case of fast reopening where the voltage is instantaneously switched to zero, the restoring elastic force immediately becomes dominant again and, thus, the micromirrors move back to their initial open state almost instantly (within a few microseconds). In the case of slow reopening with a gradual reduction in the applied voltage, the tilt angle variation is again viable, again using different force equilibria between the electrostatic and restoring elastic forces [5,6].

## 3. Capacitance–Voltage (C-V) Measurement Setups

**Figure 6:**
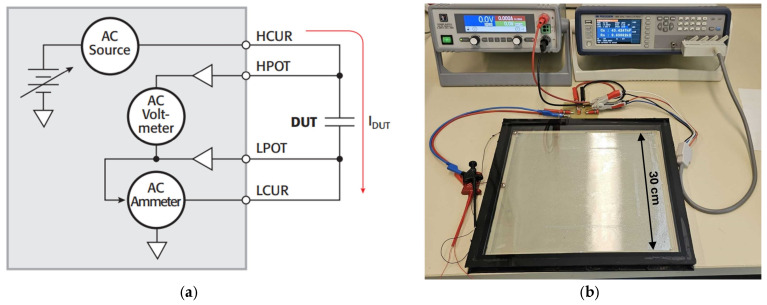
(**a**) Schematic of the LCR test system; (**b**) C-V measurement setup connected with a mounted 30 × 30 cm^2^ micromirror module. (**a**) Original from [29], with the permission of Leuze Publishing House.

For the semiconductor device and MEMS device, C-V measurements reveal three different options: classical AC impedance studies, RF technology measurements, or quasi-static capacitance studies [29,30,31,32]. The first option is based on AC impedance capacitance meters (commonly referred to as LCR meters) and is used in this work. The LCR meter measures complex impedance using an auto-balanced bridge that maintains a virtual AC ground on the sensitive side of the capacitor. This type of meter typically has a frequency range from 1 kHz to 10 MHz. LCR meters measure AC impedance by applying AC voltage from the high-current terminal (HCUR). The low-current terminal (LCUR) measures the current flowing through the device, while the high- and low-potential terminals (HPOTs and LPOTs) measure the voltage across the device, as shown in Figure 6a [29,33].

Figure 6b presents a 30 × 30 cm^2^ micromirror array module under test conditions, built as a double insulation glazing device. The space between the two glass panes is filled with argon, chosen for its inert properties and low heat conductivity. This inert gas atmosphere is essential to prevent oxidation and moisture ingress, crucial for preserving the reliability of the micromirrors as well as their electrostatic actuation. Such protection not only enhances operational stability but also extends the lifespan of the micromirror arrays considerably [28,34]. The edges of the glass are polished to prevent any potential damage to other components and to ensure a perfect seal, crucial for maintaining the integrity of the argon-filled environment. Maintaining such an inert gas environment is also very beneficial during C-V measurements, as it ensures consistent and reliable testing conditions [28]. The devices in Figure 6b at the top right and top left of the micromirror module are the LCR meter (B&K Precision Corp., Yorba Linda, CA, USA. “500 kHz/1 MHz Precision LCR meter, Models 894 & 895) and the DC power supply (EA-PS 3000 C 160 W–640 W, EA ELEKTRO-AUTOMATIK GMBH, Viersen, Germany), respectively. The test micromirror modules vary by size (e.g., 10 × 10 cm^2^, 30 × 30 cm^2^, etc.), design shape (e.g., rectangular regular apertures, irregular freeform apertures, ring-shutter arrays, butterfly designs, and so on), and by material combinations (including the deposition of additional layers, like germanium [9,35,36]). The test parameters are listed in Table 1. Measurements were conducted across a large voltage range, revealing a maximum span from −100 V to 100 V, to encompass the potential feasible range of actuation voltages. In industry and research laboratories, C-V measurement is an excellent tool to investigate the closing and reopening phenomena. It is indispensable for gaining insight into the electrical properties and behaviors of MEMS devices, particularly in the context of device miniaturization and high-precision applications. From an engineering perspective, engineers use these data to perform failure mode analysis, identify subtle defects in the manufacturing process that may affect device reliability and efficiency as well as weak potential links or degradation in materials and processes, optimize device performance, increase process efficiency, improve manufacturing processes, and ultimately increase production yield [29,32,33].

## 4. Capacitance–Voltage (C-V) Measurement Results

In this section, the results of C-V measurement profiles on different micromirror array designs with various hinge structures (designs: A, B), different initial opening angles (designs: A, C), and different materials are presented (designs: A, D). All samples used in the experimental part of this paper are 10 × 10 cm^2^ large, in order to maintain the best comparability between the corresponding C-V values.

### 4.1. Technological Fabrication and Introduction of the Four Designs Used in the Experiments

Glass substrates were coated with optically transparent and electrically conducting FTO (fluorine-doped tin oxide) layers, serving as the first electrode. SiO_2_ isolation layers were deposited on top of the FTO layers by applying PECVD (plasma-enhanced chemical vapor deposition). Next, the first photoresist layer was applied using spin coating. UV exposure of the resist through a bi-layered photomask (revealing structures on both sides on photomask [37] by a SÜSS Microtec MA6 mask aligner was carried out, followed by subsequent developing and baking to structure the photoresist via photolithography. Multiple metal layers (Al, Cr) were deposited on the resist and the SiO_2_ layer using electron beam evaporation. The metal layers serve as the second electrode. To achieve flat micromirror blades, a second lithography step was applied to deposit a further stress-compensating metal layer outside the hinge ranges. Outside of the hinge areas, the micromirrors exhibit flatness, which is required for daylight steering enabling their applicability in future energy-saving systems. After finishing the second deposition processes, the sacrificial photoresist layers below the micromirror blades and the hinges were removed. Finally, the MEMS structure was dried. These steps culminated in the successful fabrication of micromirrors with 90° or 130° opening angles.

**Figure 7:**
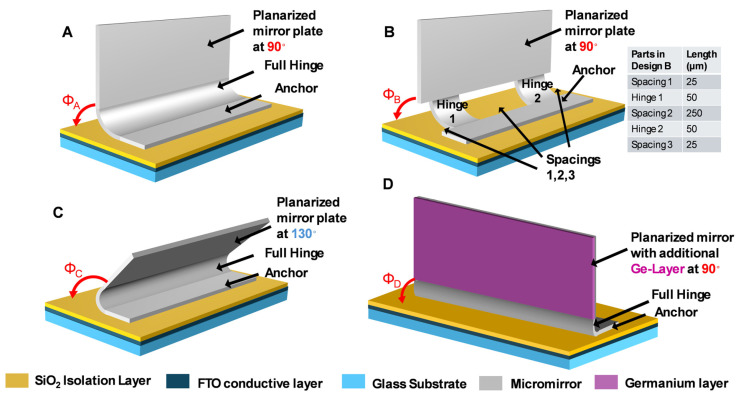
Schematics of different micromirror designs used in the experimental study. (**A**) Micromirror with a full-hinge design and an opening angle of 90°; (**B**) micromirror with a broken-hinge design, with a special configuration (spacing 1 + hinge 1 + spacing 2 + hinge 2 + spacing 3) in the hinge axis direction and an opening angle of 90°; (**C**) micromirror with a full-hinge design and a large opening angle of 130°; (**D**) micromirror comprising Al, Cr, and Ge.

Now, the four micromirror array designs which were studied via the C-V measurements are introduced. These designs are designated A, B, C, and D, and the corresponding different geometrical and material parameters are listed in Table 2 and displayed in Figure 7. It is important to note beforehand that all the given parameters in Table 2 have a significant impact on the actuation behavior, which can be interpreted from the obtained C-V curve.

**Figure 8:**
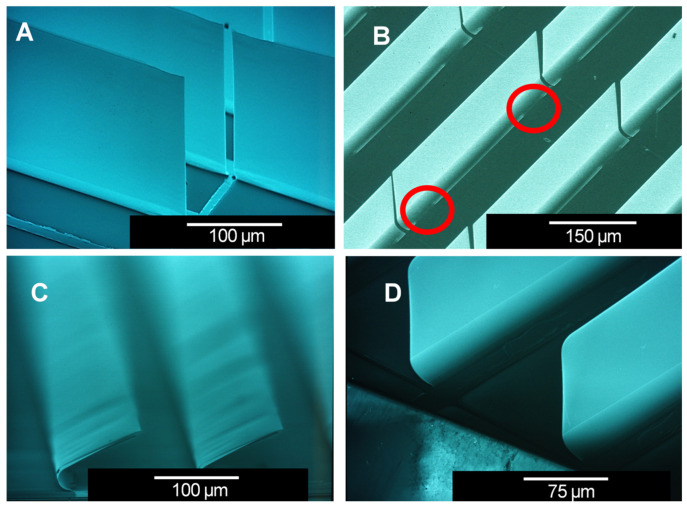
SEM micrographs of design (**A**) with a full hinge at the 90° initial opening angle, design (**B**) with broken hinges at the 90° tilt angle (broken hinges are highlighted (red circles)), design (**C**) with a full hinge at the 130° tilt angle, and design (**D**) with a full hinge at the 90° tilt angle, using the Al, Cr, and Ge layer system. Image (**A**) is an original from [18], with the permission of Leuze Publishing House. Image (**D**) is an original image from [35], with the permission of Leuze Publishing House.

Figure 7 illustrates four different micromirror designs (A–D) with differences in the designed hinge structures, initial tilt angles, and materials. All the micromirror planes are fully planarized by layer combinations with thicknesses of a few hundred nanometers. Design A is the standard design with a full hinge along its length, whereas design B has a broken hinge design feature with two short, separated hinges (hinge 1, hinge 2) that connect the mirror to the anchor. Both of the initial tilt angles Φ_A_ and Φ_B_ are designed to be approximatively 90°. Design C has the same full-hinge design as design A, but with a larger tilt angle Φ_C_ of about 130°. Designs A, B, and C are fabricated using identical thicknesses in the metal layer stack (Al, Cr). The last design, design D, has exactly the same parameters as the that of A, except with a different material combination of Al, Cr, Ge (see [35] for details). The SEM micrographs of all four designs are shown in Figure 8.

**Figure 9:**
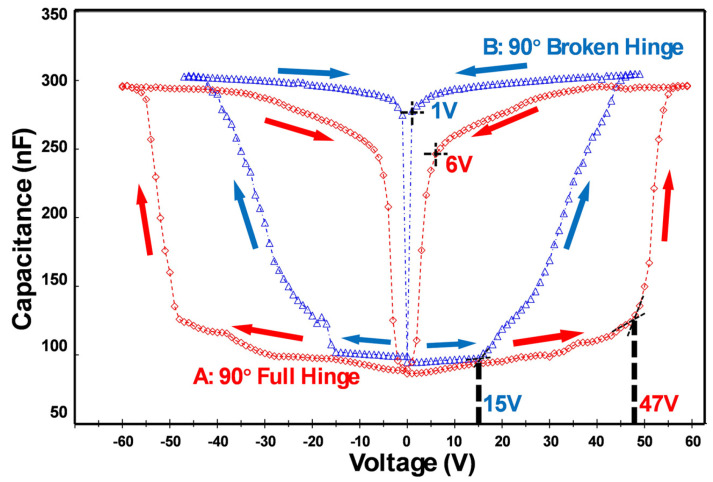
C-V curves of design A (full-hinge design with a 90° initial opening angle, red color) and design B (a broken-hinge design with a 90° initial opening angle, blue color).

Micromirror array design A is set as the reference for this study. In design B, maintaining the metal layer thicknesses inside the hinge and applying hinge interruptions (in short: broken hinge) does not vary the initial opening angles but does reduce the required threshold actuation voltage. Lower threshold actuation voltages result in lower power consumption at the end (this will be discussed in this section later; see Figure 9). The geometrical parameters of the broken hinge are symmetric in the direction of the hinge axis (see also Figure 7B). There are two 25 µm outer spacings (spacings 1 and 3) and one 250 µm central spacing (spacing 2) without hinges, respectively, and two 50 µm-long hinge parts (hinge 1 and hinge 2), which have been highlighted with two red circles in Figure 8. This provides a length of 400 µm in total, which is the same as the length of the other full-hinge structures (A, B, and D).

Design C is included to demonstrate the advantage of larger initial opening angles to enhance the possibilities and flexibility of daylight steering.

Design D improves the safety outside of the building (less glare effects in traffic) by including an additional Ge layer on one side of the mirror blades. This demonstrates that a 90° initial opening angle can be obtained with an additional Ge layer and that the functionality of light steering inside the room is still possible. We published several possible options for coating materials (not only Ge) with more details in [35].

### 4.2. C-V Measurement Results

Comparisons of the C-V study profiles for the four designs (A, B, C and D) are presented in this section.

#### 4.2.1. Comparison of Micromirror Designs A and B

The C-V curves of both micromirror array design A with a full-hinge structure and B with a broken-hinge structure are shown in Figure 9 for comparison purposes. As the applied voltage increases in the closing process, the micromirrors gradually tilt towards the substrate, resulting in a gradual increase in capacitance value. Once the applied voltages of the micromirror arrays (A, B) exceed their threshold voltage of closing V_clo,th_ (47 V and 15 V, respectively), the slope dC/dV drastically increases. The capacitance is at its maximum value when all micromirrors on arrays are in a closed state. Further increments of voltage beyond this point do not result in a further increase in capacitance. It can be seen from Figure 9 that the capacitance profile of micromirror array A (red color) rises much faster than that of micromirror array B (blue color), which indicates that the actuation of the micromirror with a full hinge is more drastic than that of the micromirror array B, which has a broken hinge.

In practice, the C-V profile of B after the V_clo,th_ (15 V) increases gradually, since the micromirrors do not close fully simultaneously. The slope dC/dV of the C-V curves represents the average capacitances of the mirrors and the voltage ranges of the closing phase. By design, the hinge length of the micromirror contributes the most to the elastic force due to the residual stress in the metal layer stack, so shorter hinges result in smaller elastic forces, which, in turn, require lower electrostatic forces for actuation. By comparing the C-V curves for A and B, it can be inferred that a micromirror design with a full hinge behaves more uniformly, as discussed in [38,39,40].

#### 4.2.2. Comparison of Micromirror Designs A and C

**Figure 10:**
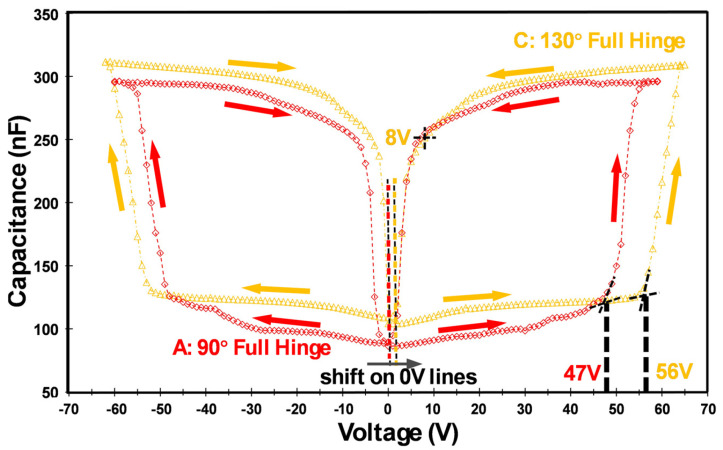
C-V curves of design A (full hinge with a 90° initial opening angle, red color) and design C (full hinge with s 130° initial opening angle, orange color); the shift of axis is shown with orange/red dotted lines.

The C-V profiles of micromirror arrays A and C are displayed in Figure 10. Considering the closing process, first, the capacitances of A and C increase moderately when the applied voltages increase. After the voltages on arrays A and C reach their threshold voltages V_clo,th_ at 47 V and 56 V, respectively, the capacitance values increase more drastically. The capacitance values are at their maximum when all the mirrors have been switched into the closed state.

Next, the reopening process is considered, and the voltage is reduced continuously from the maximum to 0 V. As a result, the capacitance also decreases, moderately at first, corresponding to high capacitance values around 250–300 nF. After passing the threshold voltage V_op,th_ (8 V), the capacitance starts to decrease more dramatically. Micromirrors with larger initial opening angles require a higher voltage to reach the threshold, because the mirrors need to move across a larger angle span. Therefore, the threshold voltage V_clo,th_ for the yellow profile (initial opening angle of 130°) is larger than that for the red profile (initial opening angle of 90°), with a voltage of 56 V for yellow and 47 V for red, respectively.

Designs A and C both have full hinges and show similar “C-V profile slopes”, as indicated by the C-V curves in Figure 10, although both were fabricated with different initial tilt angles. Although the larger tilt angles provide a larger light guiding range, it will only have a small impact on the threshold voltage phenomena, since no change is made to the resulting electric field in the actuation, because the electrode dimensions are the same in designs A and C. In contrast, a distinct difference can be observed in relation to the previous comparison between designs A and B (exhibiting different C-V profile slopes due to the different hinge dimensions, which resulted in different counteracting elastic forces). This complex interaction between counteracting elastic force and electrostatic force contributes to the differences in C-V profiles.

For the sample with a 130° initial tilt, the shift of the symmetry axis of the C-V profile is visualized via a broken black–yellow line. The shift has to be measured in relation to the 0 V point. The shift originates from the charging effect of the SiO_2_ layer. During the electrostatic actuation, charge carriers can be captured by traps in the SiO_2_ layer and thermally reemitted again later. Charging effects are often observed in semiconductor devices including insulators, and have been investigated and discussed many times [26,41,42,43].

#### 4.2.3. Comparison of Micromirror Designs A and D

**Figure 11:**
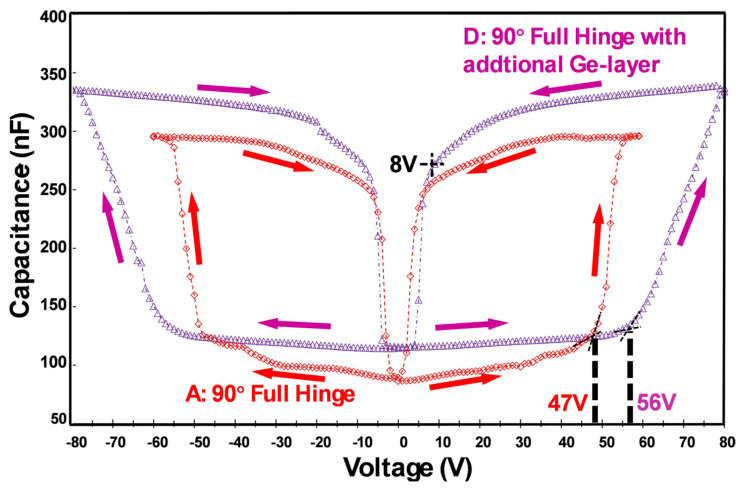
C-V curves of micromirror design A (full hinge with 90° initial opening angle, in red) and micromirror design D with an additional germanium layer (full hinge with 90° initial opening angle, in purple), respectively.

Compared to the metal layer stack used for micromirror design A, the micromirror arrays in design D are coated with an additional germanium (Ge) layer. MEMS micromirror arrays with one-sided Ge-coated mirrors result in a much smaller reflection of sunlight from the building to the outside environment than designs without Ge, which use pure metallic mirrors. This reduces the glare caused by potential sunlight reflections on car drivers and enhances safety considerably. Micromirror arrays can be fabricated with different metallic colorings (visible from outside of the building when the mirrors are closed) via varying the thickness of the additional Ge layers. The different metallic color impression details are included in Ref. [35]. The ongoing investigations of this new metal layer stack is another big topic and will be presented in a forthcoming publication. Notwithstanding this, the additional Ge layer in design D could affect the overall actuation behavior; hence, C-V measurement (Figure 11) is necessary to investigate the change in actuation characteristics, if one is present.

Note that the remaining parameters are kept the same as in designs A, B, and C. In Figure 11, the capacitance values of design D reveal a threshold voltage V_clo,th_ of 56 V. Considering the reopening process and reducing the voltage below the threshold voltage V_op,th_ = 8 V, the slope dC/dV dramatically increases.

The desired colors require specific Ge layer thicknesses, although changes are mostly within a few nanometers. Considering that the addition of Ge results in stronger restoring elastic forces, it needs to be compensated for by a proper design in the other Al and Cr layer thicknesses. One of the most important results is that mirrors including Ge in can be (i) stress-compensated to obtain planar mirror blades, (ii) enable 90° or larger initial opening angles, (iii) hysteresis in a total closing/reopening cycle is also obtained, and (iv) the electrostatic actuation functionality can be obtained, as in the other designs. Apart from that, it is worth mentioning that the ongoing work in this field has already considered how to integrate broken-hinge designs for this particular material system with Ge, which has resulted in comparable threshold voltages V_clo,th_ and V_op,th_ to that of design A. More details on this topic will be published in a forthcoming publication. The following Table 3 summarizes all the threshold voltages for the closing and reopening processes of designs A, B, C, and D.

## 5. Conclusions and Outlook

In this research, capacitance–voltage (C-V) measurement techniques were utilized to characterize micromirror arrays concerning their electrostatic actuation. Different hinge configurations and the additional integration of a Ge layer onto the metallic heterostructure stack have resulted in distinct changes to the C-V profiles, including the prominent threshold voltages of the closing/reopening processes (V_clo,th_/V_op,th_).

(i) Maintaining the metal layer thicknesses inside the hinge and applying a broken hinge does not vary the initial opening angles but reduces the required actuation voltages. Aiming at lower energy consumption of the MEMS system in building windows, broken-hinge designs are superior in comparison to full-hinge designs. The broken-hinge structure B demonstrated a lower elastic restoring counterforce, which resulted in a lower threshold voltage V_clo,th_ of only 15 V in the C-V measurement. In contrast, micromirror arrays with the full-hinge structure A require a higher threshold voltage of 47 V. (ii) Arrays with an initial angle of 130° demonstrate a larger steering angle span than arrays with an initial angle of 90°. Via adequate stress tailoring in the hinge, the feasibility of larger initial opening angles has been demonstrated. The enlarged angle range provides a larger steering angle span and more degrees of freedom in light steering. All sections of a room can be reached with daylight steering, and personalized light can be supplied independently of the user’s position. However, the designs with larger initial opening angles show a slightly higher power consumption since larger threshold voltages for the closing process are required. Also, energy consumption can be reduced by utilizing broken-hinge designs from the concept of design B. The C-V profiles from broken-hinge designs indicate huge potential to further reduce the high threshold voltage V_clo,th_, which is required for micromirror arrays with larger initial opening angles. (iii) Adding an additional Ge layer into the micromirror design D can show the full actuation performance, like that in design A. Applying MEMS micromirror arrays in building facades that switch between an uncoloured glass impression and a distinct metallic color is feasible. For future investigations, introducing the broken-hinge photomask in lithography process (as in design B) could further reduce the V_clo,th_ of design D, achieving lower energy consumption. The C-V measurements provide valuable insights for the optimization of designs, crucial for the future development and improvement of MEMS micromirror arrays for various applications.

Additionally, special rapid aging tests have been performed, such as extreme UV light exposure and elevated climate chamber temperatures in parallel, validating the durability and functionality of micromirror arrays under harsh operational conditions. Multiple reliability tests have been performed for design A. No mirror failures were observed after 53 billion electrostatically actuated open–closed–open cycles at 4 kHz and multiple temperature cycles between 0 °C and 80 °C. The MEMS arrays also survived extreme temperatures of −80 °C and 120 °C without a change in the C-V profiles. Externally excited vibrations at 3248 Hz over 36122 h did not result in failure in Supplementary Materials [44,45]. Exposure to UV radiation ten times stronger than the level which is typical for large cities in the world was performed for more than one month without an influence on the metallic MEMS being observed in Supplementary Materials [46]. These reliability tests confirm our methodology, and the results coincide with sustainable green building goals.

## 6. Patents

H. Hillmer, G. Xu: Lichttechnisches Modul für eine Gebäudefassade, EP 4102024B1 (2024).

B. Khudhair, V. Viereck, H. Hillmer: Microstructure and method of producing a microstructure in a photolithography technique. DE102015117556A1 (2017).

## Figures and Tables

**Table 1:** C-V characterization measurement conditions.

Parameters	Setting
Measurement mode	Cs (series capacitor)
Sweep voltage [V]	−100 to ~100
Stepsize (increment) [V]	1
Frequency [kHz]	10
Micromirror array size [cm^2^]	100 (10 × 10), etc.
Temperature [°C]	20

**Table 2:** Different designs of micromirror arrays (all 10 × 10 cm^2^ in size) have been studied via C-V measurements. The broken hinge design B is symmetric in the direction of the hinge axis and reveals two 50 µm-long hinge parts, whereas for the full-hinge structures (A, C, and D) there is only a single hinge of 400 µm length.

Micromirror Array Designs	Material Combinations	Hinge Difference Configurations (Spacing 1 + Hinge 1 + Spacing 2 + Hinge 2 + Spacing 3)	Opening Angle
A	Al, Cr	(0 + 400 + 0 + 0 + 0) μm	≈90°
B	Al, Cr	(25 + 50 + 250 + 50 + 25) μm	≈90°
C	Al, Cr	(0 + 400 + 0 + 0 + 0) μm	≈130°
D	Al, Cr, Ge	(0 + 400 + 0 + 0 + 0) μm	≈90°

**Table 3:** Experimental threshold voltages V_op,th_ and V_clo,th_ of designs A–D.

Micromirror Array Designs	A	B	C	D
Threshold voltage for the closing processes (V_clo,th_)	47 V	15 V	56 V	56 V
Threshold voltage for the reopening process (V_op,th_)	6 V	1 V	8 V	8 V

## Data Availability

The original contributions presented in this study are included in the article/Supplementary Materials. Further inquiries can be directed to the corresponding author.

## Supplementary Materials

The following supporting information can be downloaded at: https://www.mdpi.com/article/10.3390/mi16020157/s1, Figure S1: Experimental setups and results.
