# Supplementary material for: Capacitance–Voltage Studies on Electrostatically Actuated MEMS Micromirror Arrays
Source: Micromachines (Basel). 2025 Jan 29;16(2):157. doi: 10.3390/mi16020157 (PMC11857267; doi:10.3390/mi16020157)
Supplement: Supplementary file 1 [file micromachines-16-00157-s001.zip › micromachines-3372706-supplementary.pdf]

Supplementary File:

During the reliability studies the proper functionality of the MEMS arrays has been measured and checked from time to time.

In case of the mechanical vibration tests optical micrographs have been taken from time to time. Before the reliability tests started, a characteristically defect micromirror a characteristic group of two defect mirrors had been selected inside the array. These characteristic patterns enable to easily find again the identical positions within the otherwise monotonously looking arrays, typically every week. To take the optical micrographs, a short interruption of the aging test is required. No micromirrors were observed getting defected. The mirrors being defect from the beginning also stayed in the geometrical situation/shape as they were before the tests. For details and some example photos see Ref [44,45]

In case of the amplitude modulation response studies, the values of the maximum and minimum transmission values are compared every week without interrupting the measurements. In addition, CV measurements have been performed from time to time to identify possible deviations and possible aging. In that case an interruption of the aging test is required. No changes had been observed.

In case of exposing the arrays to extreme UV radiation combined with temperature cycles, the climate chamber has through-puts for electrical metrology cables. This allows to measure CV profiles whenever required without interrupting the aging test. No changes had been observed.

In case of multiple fast temperature cycles between 0°C – 80°C - 0°C – 80°C … another climate chamber was used also having through-puts for electrical metrology cables. This allows to measure CV profiles whenever required without interrupting the aging test. No changes had been observed. For details see Ref [46].

In case of the extreme temperatures of -80°C and +120°C, CV profiles have been recorded before and after the extreme temperature period. Also here no changes had been observed.

All tests involving very high temperatures are called rapid-aging tests.

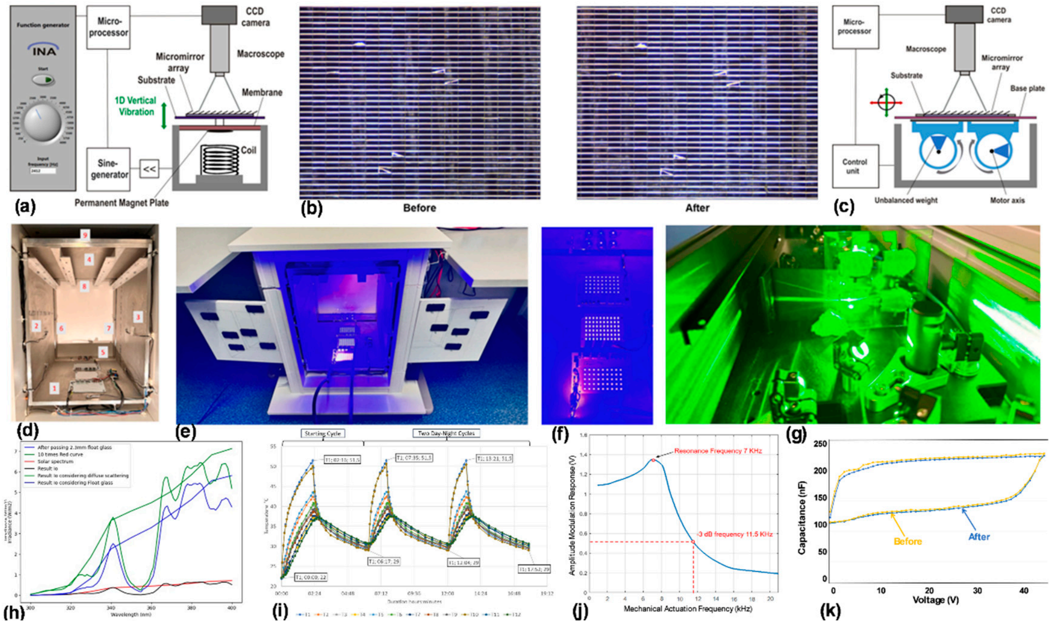

Figure S1: Experimental setups and results.

Fist row: (a) 1D mechanical vibration set-up allowing 0-10KHz vibration at small amplitudes. (b) Optical micrograph of a sample having been selected with characteristic defects to allow identification of the same sample positions before and after the aging tests. (c) 2D mechanical vibration set-up allowing 0-100Hz vibration at large amplitudes.

Second row: (d) Open UV aging chamber with the positions of 9 temperature sensors. (e) Open UV aging chamber with UV LED arrays of different wavelengths. (f) Detail representation of some UV LEDs of different wavelengths. For the aging the UV chamber had been closed also with a sixth heat insulated wall. (g) Setup measuring the aging during exposure to 10W continuous power laser radiation for 20s. The laser beam diameter was 2.3 mm.

Third row: (h) Irradiance spectra of the solar UV spectrum simulated by different UV LEDs. The red irradiance spectrum corresponds to the situation in a large city with the highest UV radiation in the world. The green and blue irradiance spectra corresponding to the ten-times higher values chosen for the rapid aging tests. (i) Temperature values measured by the 9 temperature sensors in the UV chamber during the cycles. UV LEDs produce a lot of heat. Their optical luminance yield is only between 1% and 10% of the electrical input power. The optical luminance yield is strongly reducing with decreasing wavelength. Thus, the chamber is self-heated via the UV. The heating phases show positive slopes (with LEDs switched on) and the cooling phases LEDs show negative slopes (with LEDs switched off). However the chamber also has an additional tuneable heating plate and tuneable cooling fans which have not been active for this figure. (j) Amplitude modulation response of MEMS micromirror arrays under electrostatic actuation with variable external excitation frequency. (k) Example of CV curves measured under identical conditions after some experimental cycles of rapid aging.

References

44. Nazemroaya, S.; Iskhandar, M.; Hasan, M.K.; Li, Q.; Hillmer, H. Concepts for clear View through 3D Structured Surfaces o MEMS Smart Glass: Design, Implementation, Characterization and Validation. In *Jahrbuch Oberflächentechnik*; Leuze Verlag: Bad Saulgau, Germany, 2020; Volume 76, pp. 101–115, ISBN 978-3-87480-364-9.

45 Iskhandar, M.S.Q.; Al-Qargholi, B.; Khan, M.M.; Tatzel, A.; Luo, H.; Nazemroaya, S.; Li, Q.; Hillmer, H. Development of optical MEMS-based micromirror arrays for smart window applications: Implementation of subfield addressing and reliability measurements. In *Jahrbuch Oberflächentechnik*; Leuze Verlag: Bad Saulgau, Germany, 2019; Volume 75, pp. 93–107, ISBN 978-3-87480-357-1.

46 Baby, S.; Iskhandar, M.S.Q.; Hasan, M.K.; Liebermann, S.; Chen, J.; Qasim, H.; Liu, S.; Farrag, E.; Löber, D.; Ahmed, N.; et al. Advancements in MEMS Micromirror and Microshutter Arrays for Light Transmission Through a Substrate. *Micromachines* **2025**, *16*, 103.
